# Esters in the Food and Cosmetic Industries: An Overview of the Reactors Used in Their Biocatalytic Synthesis

**DOI:** 10.3390/ma17010268

**Published:** 2024-01-04

**Authors:** Salvadora Ortega-Requena, Claudia Montiel, Fuensanta Máximo, María Gómez, María Dolores Murcia, Josefa Bastida

**Affiliations:** Department of Chemical Engineering, Faculty of Chemistry, Campus of Espinardo, University of Murcia, 30100 Murcia, Spain; dortega@um.es (S.O.-R.); cmontiel@um.es (C.M.); fmaximo@um.es (F.M.); maria.gomez@um.es (M.G.); md.murcia@um.es (M.D.M.)

**Keywords:** esters, food industry, cosmetic industry, biocatalysis, immobilized lipase, batch reactor, packed-bed reactor, fluidized bed reactor

## Abstract

Esters are versatile compounds with a wide range of applications in various industries due to their unique properties and pleasant aromas. Conventionally, the manufacture of these compounds has relied on the chemical route. Nevertheless, this technique employs high temperatures and inorganic catalysts, resulting in undesired additional steps to purify the final product by removing solvent residues, which decreases environmental sustainability and energy efficiency. In accordance with the principles of “Green Chemistry” and the search for more environmentally friendly methods, a new alternative, the enzymatic route, has been introduced. This technique uses low temperatures and does not require the use of solvents, resulting in more environmentally friendly final products. Despite the large number of studies published on the biocatalytic synthesis of esters, little attention has been paid to the reactors used for it. Therefore, it is convenient to gather the scattered information regarding the type of reactor employed in these synthesis reactions, considering the industrial field in which the process is carried out. A comparison between the performance of the different reactor configurations will allow us to draw the appropriate conclusions regarding their suitability for each specific industrial application. This review addresses, for the first time, the above aspects, which will undoubtedly help with the correct industrial implementation of these processes.

## 1. Introduction

In 1850, scientist Alexander William Williamson accidentally discovered the synthesis of ethers based on the reaction between an alcohol and an alkyl iodide in the presence of sulfuric acid [1]. Since then, esters have been recognized as one of the most crucial organic compounds for industrial applications, with uses in fields, such as food production, cosmetics, lubricants, pharmaceuticals, biodiesel additives, and various others [2]. The formation of an ester through the reaction of an alcohol and an organic acid has been a topic of great interest amongst scientists since the beginning, and it is widely regarded as the most effective method for studying the catalytic activity of acids because of its precision, ease of development, and reversibility [3].

Esters can be synthesized by means of esterification between acids and alcohols, as well as through transesterification, alcoholysis, or acidolysis reactions. Although the classical methods have been widely studied, the catalysts used have been refined and optimized over time to facilitate more efficient, productive, and eco-friendly procedures [4]. However, these traditional procedures often generate hazardous byproducts, have a considerable environmental impact, and require high energy consumption. To address these issues, enzymatic biocatalysis has been proposed as a revolutionary advancement in the biotechnology industry that demonstrates potential as a sustainable alternative to conventional processing methods for a wide range of everyday products. This method employs enzymes instead of traditional chemical catalysts to increase reaction rates [5]. Enzymes possess numerous properties that render them highly intriguing as they are the most effective catalysts in nature and operate under exceedingly mild conditions, including low pressure and temperature. Therefore, biocatalytic industrial processes for ester synthesis are classified under the “Green Chemistry” category since they comply with many of its “12 principles” [6].

Lipases (triacylglycerol hydrolases EC.3.1.1.3) are widely used enzymes in biocatalysis. Their biological function is to hydrolyze triglycerides and generate free fatty acids and glycerol, but they are known for their broad specificity. This is due to their ability to accept a variety of substrates besides glycerides, including amides. Therefore, lipases are employed in vitro to catalyze various reactions beyond their natural hydrolytic function. These reactions include esterification, acidolysis, interesterification, transesterification, aminolysis, and perhydrolysis, as well as a range of promiscuous reactions [7]. Lipases are highly stable, making them a suitable choice for use in diverse reaction media, including not only aqueous environments but also organic solvents, ionic liquids, supercritical fluids, and deep eutectic solvents (DESs) [8,9].

Lipases can be sourced from animals, plants, and microorganisms. Microbial lipases are the most valuable type when compared to those derived from plants or animals. This is due to the range of catalytic activities, the high production yield, the ease of genetic manipulation, the absence of seasonal fluctuations, the consistent supply, greater stability, and notably, the rapid growth rate of microorganisms in cost-effective culture media, such as byproducts from other industries [10]. Among the bacterial lipases that are commonly utilized in the industrial sector, *Candida antarctica* lipase B (CALB) is the most extensively used enzyme with the largest number of patents. *Candida rugosa* lipase (CRL), another significant yeast lipase, is a blend of different isoforms that is commercially available and documented as “generally recognized as safe” (GRAS) for use in the food industry [11]. Phospholipases from *Fusarium oxysporum*, *Thermomyces lanuginosus*, *Aspergillus niger*, and *Trichoderma reesei* are also employed in different industries [12].

Given the high cost of enzymes, the ability to reuse the biocatalyst is a desirable benefit that is made possible through immobilization, which also has the added benefit of improving enzyme stability. Enzyme immobilization is commonly practiced to modify and improve enzyme properties, including specificity, activity, and kinetic parameters. Moreover, the immobilization of enzymes results in improved separation from the reaction medium and promotes their reuse. Various methods for enzyme immobilization have been proposed, but ongoing research aims to find simpler and more cost-effective routes to obtain immobilized derivatives for industrial applications. Adsorption and covalent binding are both commonly used methods for immobilizing lipases onto a support material. Adsorption is a rapid and straightforward technique that typically results in minimal structural changes in the lipase, as the interactions between the enzyme and support are weak. In contrast, covalent binding induces strong interactions between the enzyme and support, which reduces the risk of enzyme desorption [13].

The availability of several commercial preparations on the market with exceptional properties, in terms of activity and stability, supports the use of immobilized lipases on an industrial scale. Undoubtedly, the most used immobilized lipase is Novozym^®^ 435, a derivative of *Candida antarctica* lipase B (CALB), which has been on the market since 1992 and is marketed by Novozymes (Bagsvaerd, Denmark). The immobilizing support is Lewatit VP OC 1600, which is a macroporous acrylic polymer resin onto which CALB is adsorbed through interfacial activation [9]. Novozym^®^ 435 does not aggregate, which would cause the loss of active sites in the enzyme, and has good stability over a wide pH range, especially in alkaline media. Furthermore, one of the characteristics of this enzyme is its ability to function in non-aqueous media (organic solvents or even solvent-free conditions) as it requires only a minimal aqueous layer to maintain its enzymatic activity. In addition, it has been reported in the literature that this thermophilic lipase can operate at temperatures above 100 °C and maintain activity even at 150 °C [14,15].

Lipozyme^®^ RM IM, also produced by Novozymes, is a widely used lipase in the industry that comes from *Rhizomucor miehei* and offers higher conversion efficiency at lower temperatures compared to other biocatalysts. It possesses unique characteristics that allow it to cleave sn-1,3 bonds, has great stability, and has high activity even with a low water content, making it suitable for use in reactions involving organic solvents. Novozymes has also marketed Lipozyme^®^ TL IM, which is a specific lipase derived from *Thermomyces lanuginosus*. It is highly effective for rearrangement reactions, such as interesterification, especially at positions 1 and 3 of triglycerides. This lipase also exhibits thermophilic properties, as it maintains suitable activity at high temperatures, up to 65 °C [5].

At present, Novozymes has restructured its product range and renamed its commercialized enzymes. Under the “Fine Chemicals” category, they now offer three immobilized lipases: Sustine^®^ 110 IM (formerly known as Novozym^®^ 435), Sustine^®^ 120 IM, and Sustine^®^ 130 IM (both lipases specific for 1,3-positions). In addition, four other immobilized lipases are available in the “Oils and Fats” section: Lipozyme^®^ 435, Lipozyme^®^ TL IM, Lipura^®^ Flex, and Lipura^®^ Select, the latter being specific for 1,3 positions. Unlike what was common on the Novozymes website, they currently do not specify the origin of their immobilized lipase preparations (www.novozymes.com/en/products, accessed on 2 January 2024).

In addition to Novozymes immobilized lipases, other commercial preparations, such as Chirazyme L-2 (from *Candida antarctica*) or Amano Lipase PS (from *Burkholderia cepacia*), are also used in ester synthesis.

Even though the first studies on ester synthesis with an immobilized lipase date back to the mid-20th century, there are very few studies that explicitly reference the reactor used in this process (geometry and configuration, operating conditions, etc.), with the majority focusing almost exclusively on batch reactors (BRs). There are even fewer studies found that describe the use of other types of reactors, such as packed-bed reactors (PBRs) or fluidized bed reactors (FBRs) [16]. Furthermore, there is a lack of research on the development of mathematical models for reaction kinetics and reactor design.

Therefore, in this present study, a systematic compilation of literature published in recent years (since 2000) is carried out, describing the reactor used in the synthesis of esters with immobilized lipases, considering the influence of the reactor configuration on the achieved conversion, as well as exploring the potential use of alternative reactors different from the conventional ones (such as membrane reactors, microreactors, etc.). Given the high number of papers found (>4900 in WOS based on the search terms “lipase” + “ester synthesis” between 2000 and 2023), in this review, only the articles that describe the enzymatic synthesis of esters used in the food and cosmetic industries have been considered. These two industries are the main ones involved in producing high-purity compounds that can be labeled as “natural”. Additionally, the processes align with the principles of “Green Chemistry” making them environmentally sustainable. Other important industrial sectors that also use esters in the formulation of their products are the biodiesel and biolubricant industries. In these cases, although the purity of the compounds used is not a primary factor when commercializing them, the growing interest in the development of sustainable processes has led to the publication, in recent years, of many studies. For this reason, this part of the study deserves to be dealt with in adequate depth in another review.

## 2. Reactors Used in the Biocatalytic Synthesis of Esters with Application in the Food Industry

The significant expansion of the food and beverages industry worldwide plays a pivotal role in propelling this market. Revenue in the food market for 2023 is estimated at US $9.36 trillion, with a projected annual growth rate of 6.74% (CAGR 2023–2028). The largest segment within this market is confectionery and snacks, accounting for a market volume of US $1.66 trillion in 2023, according to data sourced from Statista (https://www.statista.com/outlook/cmo/food/worldwide, accessed on 2 January 2024). The rising consumption of packaged food products and beverages across the globe, owing to evolving dietary patterns among the population, is expected to further boost the demand for additives used during food processing to enhance quality and nutritional content. The global food additives market had a valuation of US $98.40 billion in 2022 and is anticipated to experience a compound annual growth rate (CAGR) of 5.8% from 2023 to 2030, based on data from Grand View Research (https://www.grandviewresearch.com/industry-analysis/food-additives-market, accessed on 2 January 2024).

Esters occupy a prominent place among food additives, as they are used in a wide range of applications. Many references can be found describing the synthesis of sugar esters (used as emulsifiers, foaming agents, coating agents, or even stabilizers), aromatic esters, and even specific food additives. This industry, being regulated by strict quality standards, demands certain purities and the absence of byproducts from its products resulting from synthesis processes. For this reason, manufacturers of food additives are increasingly shifting from traditional chemical synthesis to alternative processes, with biocatalytic synthesis using immobilized enzymes being a standout method. The importance of developing new sustainable processes for the synthesis of esters used in the food industry is evident from the large number of articles found in the WOS database (>500 papers based on the search terms “ester synthesis” + “lipase” + ”food industry” between 2000 and 2023). As mentioned previously, only those papers that explicitly refer to the reactor used in enzymatic synthesis, studying various aspects, such as the influence of geometry or the configuration on the conversion achieved, have been considered. All the information collected in the database has been categorized according to the type of reactor used: tank (discontinuous and continuous), tubular (packed-bed and fluidized bed), and other types of reactors.

In Table 1 [17,18,19,20,21,22,23,24,25,26,27,28], articles describing the enzymatic synthesis of esters with an immobilized lipase in tank reactors are compiled. Since this type of reactor is the most widely used in the chemical industry, only studies reporting the use of a tank reactor with a volume greater than 50 mL have been compiled for the purpose of this review. Numerous studies conducted in small vessels (screw cap vials, Eppendorf vials, etc.) used for preliminary investigations into the development of new products/processes have not been considered. However, it is surprising that very few studies have looked specifically at how the characteristics of tank reactors affect the outcome of the synthesis process. Moreover, these reactors are commonly used in industry, and new studies focus more on introducing other types of reactors than on improving existing ones. Out of the 30 articles found on WOS for “ester synthesis” + “lipase” + “tank reactor” + “food” during the considered period, only 12 articles were selected for this review. The table shows that the types of esters produced enzymatically in tank reactors for this type of industry are not very diverse: sugar esters [17,18,19,25], flavors [24,26], or emulsifiers [20,21,22,23,28].

It is convenient to specify that sugar esters, which are surfactants obtained from a sugar and a fatty acid, are natural ingredients widely used in detergents, cosmetics, pharmaceuticals, and the food industry. This wide applicability means that the papers about these compounds included in this review have been classified according to their application in the food or cosmetics industry as indicated by the authors in the introduction to the papers, although all of these studies could be included in either of the two sections. As for the immobilized lipase utilized, researchers primarily choose to use commercial preparations [17,18,19,23,24,26,27], with Novozymes products being the most popular option. A few papers describe and optimize the immobilization process [20,21,22,28], and only in one study, the immobilized lipase consists of a non-growth state microorganism (*Rhizopus microsporus*) adsorbed onto a porous support [25].

Due to its ease of operation, the most used tank reactor is the batch reactor, although it operates in a non-steady state, which complicates the design equations. Only two references have been found that describe continuous operation in tank reactors [17,25]. In the first one, immobilized lipase particles are confined in a stainless-steel basket, and in the second, there is no explicit reference to the procedure used to retain the solid biocatalyst within the vessel. The fact that they operate in steady state does not seem to compensate for the operational difficulties that a continuous process has. Only in a few situations is fed-batch the operational option selected. It is described in the literature that short-chain acids can provoke the deactivation of the enzyme. To prevent this phenomenon, an excess of alcohol is often used, and, while it can positively affect enzymatic activity, it also complicates and increases the cost of separation and purification operations for the final product. Therefore, the use of a fed-batch reactor has been proposed, in which the acid is fed into the reactor in successive additions until the appropriate molar substrate ratio is achieved [26].

One of the strengths of using tank reactors is their operation in complete mixing, which ensures homogeneity in the reaction medium (concentrations and temperatures). For this reason, the stirring geometry and speed are crucial facts. It is quite common for publications to not explicitly reference the type of stirrer used [17,27], so it is assumed that a magnetic stirrer is employed, which is the most common in laboratory-scale processes [18,24,26]. However, the detrimental effect of this stirring and mixing procedure on the physical structure of the solid particles of immobilized lipase [9] leads researchers to use overhead stirrers when aiming to implement these processes on an industrial scale [19,20,21,22,23,25,28]. The significance of the stirring device is highlighted in two papers where the performances of different types of agitator blades are compared and their influence on process productivity is studied [19,23].

The removal of water generated in esterification is a vitally important aspect since, if not performed correctly, it can shift the reaction equilibrium towards hydrolysis. In most processes, molecular sieves are chosen for this purpose [17,18,25,26], although the option of conducting reactions in open-air reactors to allow for water evaporation or even using N_2_ bubbling and a vacuum has also been described [20,21,22,23,27,28]. Some of the synthesized products are high-molecular-weight esters that have high viscosity, so many reactions are carried out in organic solvents, such as acetone, 2-methyl-2-butanol, and n-hexane [17,18,19,25]. However, to simplify the final product separation and purification and comply with the principles of “Green Chemistry”, many researchers choose to perform ester synthesis in solvent-free reaction media [20,21,22,23,26,28]. Special mention of the use of supercritical CO_2_ should be made [18,24], which is a trend in recent years.

The results published in the reviewed papers are very promising, and conversions over 90% are achieved in most of them [19,21,22,23,28]. In addition, the authors point out that certain operating variables must be controlled to obtain good yields, such as the quantity of molecular sieves for water removal [17], using diverse methods for water removal [20], adjusting the enzyme concentration and substrates molar ratio [18], or implementing a different operating mode (fed-batch) [26]. In summary, it can be affirmed that the use of batch reactors is the primary choice when approaching the biocatalytic synthesis of esters, and in most cases, successful results are obtained.

As mentioned above, the tank reactors are not usually chosen for continuous operation; instead, it is more common to implement tubular reactors, either a packed-bed or fluidized bed, because they provide better results achieving higher productivities per unit of reactor volume. However, the main advantage of using tubular reactors is undoubtedly that, in the absence of mechanical agitators, the immobilized enzyme particles are not damaged and can therefore maintain their catalytic capacity for a longer period.

The use of tubular reactors for ester synthesis is rather infrequent in the chemical industry and in the food industry. However, the use of this type of reactor for the biocatalytic synthesis of food ingredients has been the subject of numerous studies published in recent years. Thus, in the last 23 years, 111 articles were found in WOS using the keywords “lipase” + “ester synthesis” + “packed bed reactor”. If the search is carried out with a change in the type of reactor to “fluidized bed reactor”, an additional 15 articles will be added to the list. Among these, only 16 articles, specifically related to the food industry, are included in Table 2 [29,30,31,32,33,34,35,36,37,38,39,40,41,42,43,44]. In this case, there was no restriction on the reactor volume as all the papers refer to preliminary studies, and the reactor dimensions are relatively small, usually in the order of a few centimeters or even millimeters. Only five papers describe the use of a fluidized bed reactor [33,37,38,39,40], with the packed-bed configuration being the most common. Not all processes are continuous, as there are specific cases where part of the reaction medium is recirculated [37] or even the entire reactants are recirculated through the bed using a storage tank as a reservoir, making the operation occur in batch cycles [38,40].

The characteristics of the above-cited processes are very similar to those described in the papers gathered in the previous table. The synthesized esters are mostly sucrose esters [29,30,31,32,34,38,40], although flavor compounds [35,36,39,41,42], modified fats, and structured lipids are also described [33,37,43,44]. Only four studies report the use of lipases that are in-lab immobilized by the authors [36,37,38,39], while the majority opted for the use of commercial immobilized lipases. Moreover, many processes are conducted in solvent-free reaction media [33,34,37,38,39,43,44], whereas those using organic solvents employ acetone [29,30,31,32,40], iso-octane [35], n-heptane [41], etc. Special mention should be made of those that propose the use of supercritical CO_2_, which is a trend in recent years, as previously mentioned [36,42].

The outcomes from utilizing tubular reactors vary, although most authors agree that both packed-bed and fluidized bed reactors offer promising potential for the future due to their ease of use, scalability, affordability, and effectiveness. The growing interest in implementing these reactors on an industrial scale is evidenced by studies comparing the performance of the commonly used tank reactor with other reactor configurations, including membrane, packedbed, and fluidized bed reactors. Table 3 [45,46,47,48,49] provides a compilation of these studies.

Several studies have highlighted the advantages of the packed-bed configuration in terms of conversion and productivity, making it a preferred option for synthesizing butyl butyrate [47] and isoamyl acetate [48]. On the other hand, some experts have noted that the fluidized bed reactor exhibits better performance in the production of amyl caprylate when compared to the batch reactor [46]. The use of a membrane reactor [45] and DES in batch and tubular reactors [49] also appears to be a viable alternative for producing esters for use in the food industry.

Table 4 compiles literature [50,51,52,53,54,55,56] on enzymatic ester synthesis using non-conventional reactors.

One notable category is microreactors [52,53,54,55]. The increasing use and advancement of microfluidic systems offers promising opportunities for researchers investigating catalytic processes. The benefits of miniaturized reactors are numerous. The compact size of these systems allows for portable applications and reduces the amount of reactant consumption and required samples. Additionally, improved heat and mass transfers enhance reaction control, while smaller reaction channels and chambers enable a more in-depth analysis. Moreover, higher degrees of automation present opportunities for both industrial production and research applications [57]. However, to date, most of the devices described have been utilized in healthcare and pharmaceutical applications with minimal research focused on synthesizing products for industries outside of these fields, such as esters.

Table 4 shows the characteristics of four processes used to synthesize different esters with food applications using microreactors, both tank [52,55] and packed-bed [53,54], with excellent results, proposing an interesting alternative to traditional reactors. On the other hand, the same table shows two other studies that investigate the operability of a tank reactor with a membrane [50,51] and a tank reactor with a rotating basket operating in successive batches [56]. In both cases, the esters synthesized belong to the sucrose esters group, obtaining conversions of 93% and 80%, respectively.

## 3. Reactors Used in the Biocatalytic Synthesis of Esters with Applications in the Cosmetic Industry

Cosmetics are commonly recognized as a category of items associated with personal grooming, particularly skincare. They have a history that likely dates to the beginnings of human civilization. The widespread use of cosmetic products in daily life gained momentum with the emergence of synthetic organic chemistry, which made it feasible for people to access desired ingredients and formulations with relative ease. Today, cosmetics hold a significant place, especially considering the recent “wellness” movement. Manufacturers must continually enhance their products to maintain a competitive edge in a market where consumers expect more choices and increasingly effective solutions. It is worth noting that most cosmetic products have a shelf life of less than five years, and manufacturers reformulate approximately 25% of their products each year [58].

In 2023, the Beauty and Personal Care market was estimated to generate a revenue of approximately US $625.70 billion. This market is anticipated to experience an annual growth rate of 3.32% from 2023 to 2028 according to data published by Statista (https://www.statista.com/outlook/cmo/beauty-personal-care/worldwide, accessed on 2 January 2024). The global cosmetic ingredients market, as of 2022, had a market size of about US $32 billion. It is expected to reach approximately US $55.44 billion by 2032, with a recorded compound annual growth rate (CAGR) of 5.7% during the forecast period from 2023 to 2032 (https://www.precedenceresearch.com/cosmetic-ingredients-market, accessed on 2 January 2024). As a result, cosmetic chemicals constitute a significant sector within the chemical industry primarily served by chemical companies, like BASF, Evonik Industries, Clariant, and Rhodia. Cosmetic products, overall, are predominantly promoted by international corporations, such as Procter & Gamble, L’Oreal, Unilever, Beiersdorf, and Colgate-Palmolive [58].

The worldwide beauty industry is typically segmented into five primary categories: skincare, hair care, color cosmetics (makeup), fragrances, and toiletries. The formulation of these cosmetic products is primarily influenced by factors, like their intended use, manufacturer preferences, and the target market. Esters, among all the classes of organic compounds employed in cosmetics, have diverse applications within the cosmetic sector. They serve as emollients in creams, act as surfactants in shampoos, function as antioxidants in anti-aging creams, contribute to fragrances in perfumes, and provide flavors in lip cosmetics, based on their distinct properties [59].

At present, the industrial production of cosmetic esters involves high-temperature synthesis with either an acid or a base catalyst, requiring temperatures as high as 150–240 °C. These elevated-temperature conditions result in the production of products of inferior quality (inappropriate for skin applications) that require additional treatments and expenses. Enzymatic processes offer a compelling solution to address these challenges, as they operate at lower temperatures (30–70 °C) and lower pressures, resulting in the creation of ultrapure, colorless, and odorless products. Esters produced through biocatalysis can be considered environmentally friendly, aligning with the growing consumer demand for “green” and “natural” products [59]. This is a primary driver behind the substantial number of publications on enzymatic cosmetic ingredient synthesis. Furthermore, the strong interest in implementing these processes on an industrial scale has motivated researchers to conduct applied research using reactors of a significant volume, along with the development of kinetic and mass transfer studies to facilitate the process scale-up.

These efforts have resulted in the commercialization of several cosmetic ingredients obtained via biocatalysis. Evonik Industries AG was the first in this field and currently offers five emulsifiers synthesized through biocatalytic processes: isoamyl cocoate (Tegosoft AC MB), cetyl ricinoleate (Tegosoft CR MB), decyl cocoate (Tegosoft DC MB), myristyl myristate (Tegosoft MM MB), and oleyl erucate (Tegosoft OER MB). The company website highlights that these esters have been “produced by an eco-efficient (enzymatic) process leading to a minimized environmental footprint” (https://personal-care.evonik.com, accessed on 2 January 2024). Afterward, the Eastman Company produced 2-ethylhexyl palmitate using GEM™ technology, which, according to the website (https://www.eastman.com, accessed on 2 January 2024), is “a bio-catalytic process that uses enzymes and closely controlled manufacturing conditions to eliminate high temperatures, strong acids, and unwanted by-products, consumes less energy compared with conventional manufacturing processes”. As far as we know, only these two companies have commercialized cosmetic ingredients using enzymatic processes.

In the WOS database, a search using the terms “ester synthesis” + “lipase” + “cosmetic” for the years 2000–2003 yielded 254 papers. As mentioned in the previous section, the tank reactor appears as the primary choice for the biocatalytic synthesis of cosmetic esters, although a search in WOS with the terms “ester synthesis” + “lipase” + “tank reactor” + “cosmetic” over the last 23 years yielded only five articles. To incorporate a greater number of studies, the search parameters were adjusted by removing the term “cosmetic”, resulting in 62 papers, of which 43 studies met the established criteria (tank reactors with a volume superior than 50 mL and cosmetic applications). Table 5 [60,61,62,63,64,65,66,67,68,69,70,71,72,73,74,75,76,77,78,79,80,81,82,83,84,85,86,87,88,89,90,91,92,93,94,95,96,97,98,99,100,101,102] compiles these papers. The esters synthesized fall into these categories: emollient esters [60,61,62,65,69,70,71,73,74,79,80,81,82,83,84,85,86,89,91,92,93,94,95,96,97,98,99,100,102], fragrant esters [63,76,77,78,87,90], sugar esters [64,67,75], and derivatives of active ingredients [66,68,72,88,101].

The rise in the use of biocatalytic synthesis to produce cosmetic esters in the industry has made commercial immobilized lipases the preferred choice for potential manufacturers and researchers. This guarantees a constant supply of enzymes. According to Table 5, among 43 papers reviewed, 36 report the use of commercial lipases from different companies: Novozymes [60,61,62,63,64,65,66,68,70,72,73,74,75,76,77,78,79,80,82,88,90,91,92,93,96,97,98,99,101], Fermenta Biotech Ltd. (Thane, India) [71,81,83,84,86,89,95], and Purolite (King of Prussia, PA, USA) [80]. In five articles, researchers describe the use of an in-lab immobilized lipase [61,69,85,94,100,102], and only two studies involve microbial cells with lipase activity [67,87]. It is evident that commercial immobilized enzymes come with a high cost. However, the immobilization processes also incur significant expenses, which can potentially surpass the cost of commercial enzymes. In one of the referenced papers, an economic analysis of the synthesis process for a mixture of wax esters similar to spermaceti (used in cosmetics for extremely cold climates) was conducted. The study demonstrated that the direct production costs of one gram of this product using in-lab immobilized lipase was comparable to that obtained using the commercial lipase Novozym^®^ 435 [85], revealing that, after thorough optimization of the immobilization process, the option of using a lipase immobilized by the manufacturers themselves can be a valid alternative to commercial products, thus avoiding excessive dependence on certain production sectors.

In most of the papers compiled in Table 5, it can be observed that they chose to develop their investigations in large tank reactors, including 2 L [65] and 300 L [82], to speed up the industrial applicability of the investigations. Within them, studies are conducted to determine the most suitable agitator geometry and to address scale-up issues. For the same reason, most researchers choose batch reactors, which are easy to operate and provide very good results in terms of productivity. There are only two references in which continuous tank reactors were employed, and these make explicit mention of the procedure used to retain the solid particles of the immobilized enzyme inside the reactor: a stainless-steel basket [64] and a membrane [87]. Of particular importance are the papers describing the synthesis of cosmetic ingredients, mainly emollients, in batch reactors using ultrasound as an energy source [74,76,81,83,86,89,95]. On the other hand, the inhibitory effect of short-chain fatty acids (pKa ≤ 4.8) on lipase activity is once again shown in some of the biocatalytic processes, as previously noted in the production of esters for the food industry [26]. Thus, heptanoic [92] and caprylic acids [98] exert this inhibitory effect, which is avoided by using fed-batch reactors that maintain the acid concentration at an optimal level. The authors suggest using either successive acid additions [92] or continuous addition using a peristaltic pump [98]. In other cases, the fractional addition of alcohols, such as butanol and ethanol, is employed to prevent their potential inhibitory effect [79]. Finally, one study was found in which one of the substrates (maltose) was added in batches because of its partial solubility in the reaction medium [64].

As for the removal of water formed in the reactions, the procedures used are similar to those described in the previous section. These methods involve the use of molecular sieves [64,67,71,75,81], vacuum operation with dry N_2_ bubbling [66,69,70,72,73,80,85,91,93,97,100], and atmospheric evaporation [69,70,92,96,98,99]. Regarding the use of organic solvents as a reaction medium, solvent-free systems are becoming more prevalent [61,62,66,68,69,70,71,72,73,74,75,79,80,81,82,83,84,85,86,89,91,92,93,95,96,97,98,99,100,101,102]. This is probably due to the additional costs associated with the final separation and purification steps of the synthesized product, as well as the need to eliminate any trace of solvents from the final product, which could interfere with its use on the skin. Other alternative solvents, such as supercritical CO_2_ [60,63] and eutectic mixtures [101], have also been employed. In all cases, very high final conversions are reported, highlighting the feasibility of an enzymatic process for the synthesis of esters with cosmetic applications in tank reactors, even in cases where the obtained compounds have a high molecular weight, which could potentially complicate mass- and heat-transfer processes within the reactor.

It is evident that there is a high interest in implementing these processes on an industrial scale, which has led numerous researchers to expand their fundamental studies. This expansion involves not only using large-volume tanks but also developing kinetic models based on the mechanism of the studied reactions, which enables the design of the reactor for its future scale-up [62,65,71,74,77,78,81,84,88,90,94,95,97,102].

The high economic cost of such processes is perhaps the main drawback that opponents of biocatalytic synthesis cite as a reason not to pursue them as an alternative to traditional chemical routes. Therefore, economic studies have been conducted [85,93,98,99] demonstrating that the unit cost of these esters, when the immobilized lipase is appropriately reused, is comparable to that described in the literature for fine chemical compounds [103]. On the other hand, increasing environmental awareness is prompting manufacturers to implement sustainable processes that align with the principles of “Green Chemistry”.

However, the perception that biocatalysis is “environmentally friendly” and a “technologically robust approach from an industrial point of view” has not always been substantiated with convincing metrics. It is common to rely on somewhat empty claims regarding the ecological nature of a particular biocatalytic process [104]. For this reason, in recent years it has become popular to include the so-called “green metrics” in the development studies of biocatalytic processes, as can be seen in some of the most recent papers of those compiled in Table 5 [98,99,101]. On the other hand, only one paper [100] deals with the design and simulation of a plant for the production of an ester mixture, which, according to the authors, would produce 173.25 kg of product per working day with a purity of 99.55%. This is an avenue to be explored if these processes are to be successfully implemented on an industrial scale.

Among the 126 articles that WOS provides based on the searches “lipase” + “ester synthesis” + “packed bed reactor” and “lipase” + “ester synthesis “ + “fluidized bed reactor”, the manual screening has allowed for the selection of the 22 shown in Table 6 [105,106,107,108,109,110,111,112,113,114,115,116,117,118,119,120,121,122,123,124,125,126], which describe ester-synthesis processes with applications in cosmetics, using the packed-bed reactor in 18 of them and the fluidized bed reactor in only 4 of them, the latter corresponding to the most recent publications.

Esters synthesized in tubular reactors are used as emollients [106,108,109,113,114,115,117,120,121,122,126], fragrances [110,116,119,123], surfactants [105,107,112,118,124,125], or active ingredients [111]. The biocatalysts used are the same as those employed in the tank reactors. Noteworthy is the greater presence of in-lab immobilized lipases [110,114,118,119,120,121,125,126]. These data are important as they indicate that the authors are trying to introduce modifications to the traditional processes using the tank reactor, which is displayed not only in the attempt to avoid the use of commercial immobilized enzymes, but also in novel reactor configurations that include recirculation [107,114,119,120,124,126] or batch use with reuses [115,122]. A special configuration uses an additional column of molecular sieves to remove water from the reaction medium [110,114].

Undoubtedly, the most important factor to highlight in this group of papers is the high presence of studies that develop kinetic and mass transfer mathematical models, which are of great importance for scaling up [120,121,125,126]. In addition, there are also outstanding works in which the simulation of the industrial plant and an economic study are carried out based on data obtained on a pilot-plant scale [112,122].

During the bibliographical search, several papers have been found that are concerned with a comparative study of different types of reactors to select the most suitable one for the process under study. Table 7 shows these nine papers [127,128,129,130,131,132,133,134,135].

As can be observed, most of the papers point out that both packed and fluidized bed reactors give better results than tank reactors [127,128,129,130,132,134,135], highlighting the advantage of using tubular reactors, especially fluidized bed and bubble column reactors, since these configurations avoid the mechanical damage that stirrers can cause to the solid particles of immobilized lipase. It is very interesting to note a study that states that the best option for synthesizing kojic acid derivatives is the batch reactor, which is unusual for these type of studies [133]. In other reports, the results obtained in a conventional batch reactor are compared with those obtained in another reactor of the same geometry but equipped with ultrasound [131] or N_2_ bubbling [135]. Both give higher conversions than the batch reactor. Finally, it is also important to highlight the incorporation of microreactors in the processes for obtaining cosmetic esters, although the number of papers found is very small. Table 8 [136,137,138] shows the main characteristics of these reactors.

As can be seen, two articles describe the use of packed-bed microreactors [137,138] and the third one involves a membrane reactor with a single channel [136]. In all processes, good results are obtained showing the promising future of these type of reactors in the industry.

## 4. Conclusions

Esters are compounds of a diverse nature and structure, making them applicable in a wide variety of industrial segments. Among these, the food and cosmetic industries stand out. In recent years, these two productive sectors have become highly conscious of the use of high-purity compounds, not only to meet strict international regulations but also to satisfy the increasingly demanding preferences of consumers. In this context, biocatalysis emerges as an alternative that provides food and cosmetic product manufacturers with the indispensable esters to be used in their formulations. The mild operating conditions of enzymatic processes allow for the synthesis of high-purity compounds with almost the complete absence of undesired by-products. Additionally, biocatalytic synthesis aligns with many of the 12 principles of “Green Chemistry”, enabling products to be labeled as “natural”.

On the other hand, the significant presence of commercial immobilized lipases in the international market with high activity and stability has encouraged companies to incorporate biocatalytic processes into their production lines. However, to avoid dependence on the supply of immobilized enzymes, the lack of which could affect production, numerous studies in the literature explore innovative methods for lipase immobilization and their application in ester synthesis. The controversy surrounding the potentially excessive cost of preparing the biocatalyst compared to the high price of commercial immobilized enzymes also features prominently in the papers surveyed in this review.

It appears that manufacturers of food additive and cosmetic ingredients predominantly conduct their production using tank reactors, mostly in batch mode. For this reason, many studies aim to investigate the possibilities of applying other operation procedures (continuous) or even different reactor configurations. Numerous papers describe the successful performance of continuous reactors, both tank and packed-bed. Special mention should be made of attempts to incorporate fluidized bed reactors, which, in addition to providing satisfactory results, are particularly suitable for preventing the breakage of immobilized enzyme particles. Furthermore, in recent years, there has been emerging interest in the incorporation of new microreactors, although many studies will be necessary before their implementation on an industrial scale.

On the other hand, it is crucial to highlight the need for developing kinetic, mass-transfer, and reactor-design models, which are of decisive importance when designing and simulating a biocatalytic ester-synthesis plant. Additionally, there is a need to raise awareness among process engineers about the importance of conducting sustainability and economic studies, which are essential for the successful industrial-scale implementation of these production processes.

Finally, it is important to highlight that the introduction of artificial intelligence to the sustainable biocatalytic synthesis of esters holds significant promise. By leveraging artificial intelligence, various aspects of the manufacturing process can be optimized to improve efficiency, reduce the environmental impact, and enhance overall sustainability. AI can contribute to the design of eco-friendly processes, precise control of production parameters, and real-time monitoring, leading to more resource-efficient and environmentally friendly synthesis practices. Additionally, the integration of artificial intelligence can open new avenues for innovation and the development of novel solutions to address challenges in the sustainable production of esters for applications in various industries, such as food and cosmetics.

## Figures and Tables

**Table 1:** Biocatalytic synthesis of esters with applications in the food industry using tank reactors.

Ester	Biocatalyst	Characteristics	Reference
Monolauroyl maltose	Chirazyme^®^ L-2 C2 immobilized *Candida antarctica* lipase B	Batch and continuous stirred tank reactors Volume: 300 mL Immobilized lipase packed into a stainless-steel basket Solvent: acetone Water removal: molecular sieves Conversion: 60% after 90 h	[17]
Fructose palmitate	Novozym^®^ 435 immobilized *Candida antarctica* lipase B	Batch reactor Volume: 100 mL Solvent: 2-methyl-2-butanol, supercritical CO_2_ Water removal: molecular sieves Conversion: 78% after 72 h	[18]
Oleyl palm ester	Lipozyme^®^ RM IM immobilized *Rhizomucor miehei* lipase	Batch reactor with different impellers Volume: 2 L and scale up to 50 L Solvent: n-hexane Conversion: 97.2% after 5 h	[19]
Ricinoleic acid estolides	*Candida rugosa* lipase in-lab immobilized in Lewatit MonoPlusMP64	Batch reactor Volume: 100 mL Solvent: solvent-free Water removal: atmospheric evaporation and vacuum (comparison) Conversion: 68% after 24 h	[20]
Polyglycerol polyricinoleate	*Candida rugosa*, *Rhizopus arrhizus*, and *Rhizopus oryzae* lipases in-lab immobilized in Lewatit MonoPlusMP64	Batch reactor (two steps) Volume: 100 mL Solvent: solvent-free Water removal: vacuum Conversion: 91.5% after 125 h, 98% after 320 h	[21,22]
Polyglycerol polyricinoleate	Novozym^®^ 435 immobilized *Candida antarctica* lipase B	Batch reactor with different impellers Volume: 100 mL Solvent: solvent-free Water removal: vacuum and dry N_2_ bubbling Conversion: 99.3% after 55 h	[23]
Eugenyl acetate	Lipozyme^®^ 435 and Novozym^®^ 435 immobilized *Candida antarctica* lipase B	Batch reactor Volume: 100 mL Solvent: supercritical CO_2_ Kinetic model Conversion: 45% after 6 h	[24]
Ethyl oleate	Dry biocatalyst of supported *Rhizopus microsporus* with lipase activity	Continuous stirred tank reactor Volume: 700 mL Solvent: n-hexane Water removal: molecular sieves Conversion: 90% after 14 h	[25]
Benzyl butyrate	Novozym^®^ 435 immobilized *Candida antarctica* lipase B Lipozyme^®^ TL-IM immobilized *Thermomyces lanuginosus* lipase Lipozyme^®^ RM IM immobilized *Rhizomucor miehei* lipase NS 88011 Non-commercial immobilized *Candida antarctica* lipase B	Batch and fed-batch reactors Volume: 500 mL Solvent: solvent-free Water removal: molecular sieves Conversion: 80% after 12 h	[26]
Stearidonic acid-rich triacylglycerol	Novozym^®^ 435 immobilized *Candida antarctica* lipase B Lipozyme^®^ TL-IM immobilized *Thermomyces* *lanuginosus* lipase	Batch reactor (two steps) Volume: 50 mL Water removal: vacuum Conversion: 86.4% after 12 h	[27]
Polyglycerol polyricinoleate	*Candida antarctica* lipase B in-lab immobilized in Lewatit MonoPlusMP64	Batch reactor Volume: 100 mL Solvent: solvent-free Water removal: vacuum and dry N_2_ bubbling Conversion: 98% after 159 h	[28]

**Table 2:** Biocatalytic synthesis of esters with applications in the food industry using tubular reactors.

Ester	Biocatalyst	Characteristics	Reference
Acyl mannoses	Chirazyme^®^ L-2 C2 immobilized *Candida antarctica* lipase B	Packed-bed reactor (continuous) 10 mm i.d. × 50 mm Residence time: 12 min Solvent: acetonitrile, acetone, 2-methyl-2-propanol, 2-methyl-2-butanol Conversion: 40% for 16 days	[29]
Acyl L- ascorbates	Chirazyme^®^ L-2 C2 immobilized *Candida antarctica* lipase B	Packed-bed reactor (continuous) 4.6 mm i.d. × 150 mm Residence time: 5 min Solvent: acetone Productivity: 1.6–1.9 kg/L for 11 days	[30]
Lauroyl and oleoyl erythritol	Chirazyme^®^ L-2 C2 immobilized *Candida antarctica* lipase B	Packed-bed reactor (continuous) 10 mm i.d. × 50 mm Residence time: 4.5 min Solvent: acetone Productivity: 1.25–1.6 kg/L for 14 days	[31]
Fatty acid esters of sugar alcohols	Chirazyme^®^ L-2 C2 immobilized *Candida antarctica* lipase B	Packed-bed reactor (continuous) 20 mm i.d. × 50 mm Residence time: 15 min Solvent: acetone Productivity: 1.3–2 kg/L for 2 days	[32]
Esters of palm stearin with soybean oil	Novozym^®^ 435 immobilized *Candida antarctica* lipase B	Fluidized bed reactor (continuous) 2 cm i.d. × 20 cm Residence time: 19 min Solvent: solvent-free Conversion: 10–45% for 21 days	[33]
Monoglycerides of Babassu oil	Lipase PS—Batch number: 01022TD in-lab immobilized *Burkholderia cepacia* lipase	Packed-bed reactor (continuous) 1.5 cm i.d. × 5.5 cm Residence time: 356 min Solvent: solvent-free Conversion: 25–33% for 22 days	[34]
Farnesyl laurate	Lipozyme^®^ RM IM immobilized *Rhizomucor miehei* lipase	Packed-bed reactor (continuous) 1.2 cm i.d × 9.24 cm Residence time: 22 min Solvent: iso-octane Kinetic and mass transfer model Conversion: 98.07% for 3 h	[35]
Butyl acetate	*Candida antarctica* lipase B in-lab immobilized in porous γ-alumina pellets	Packed-bed reactor (continuous) 12 g biocatalyst Flow rate: 0.5–10 mL/min Solvent: n-hexane, supercritical CO_2_ (comparison) Productivities: 119 µmol/min × g pellets and 501 µmol/min × g pellets	[36]
Esters of milkfat with soybean oil	*Rhizopus oryzae* lipase in-lab immobilized in polysiloxane– polyvinyl alcohol particles Novozym^®^ 435 immobilized *Candida antarctica* lipase B	Fluidized bed reactor (recirculating and continuous) 20 mm i.d. × 200 mm Residence time: 12 min and 6 min Solvent: solvent-free Conversion: 52% and 27% for 190 h	[37]
Fructose oleic ester	*Candida rugosa* lipase in-lab immobilized in modified Amberlite IRA-96	Fluidized bed reactor (batch recirculating) 10 mm i.d. × 160 mm Residence time: 42.78–213.91 min Solvent: solvent-free Conversion: 197.06% (mixture of mono- di- and tri- esters) 15 cycles	[38]
Isoamyl acetate	*Aspergillus oryzae* lipase in-lab immobilized in calcium alginate beads	Gas-liquid fluidized bed reactor (continuous) 0.8 mm i.d. × 143 mm Continuous ethanol removal: N_2_ flow Solvent: solvent-free Conversion: 89.55% for 60 min	[39]
Monolauroyl maltose	Novozym^®^ 435 immobilized *Candida antarctica* lipase B	Fluidized bed reactor (recirculating) 10 mm i.d. × 300 mm Flow rate: 1 mL/min Solvent: acetone Conversion: 30% for 5 days	[40]
Geraniol esters	Novozym^®^ 435 immobilized *Candida antarctica* lipase B Lipozyme^®^ TL-IM immobilized *Thermomyces* *lanuginosus* lipase Lipozyme^®^ RM IM immobilized *Rhizomucor miehei* lipase	Packed-bed reactor (continuous) 3.0 mm i.d. × 100 mm Residence time: 5–25 min Solvent: n-heptane Kinetic model Conversion: 87% for 25 h	[41]
Isoamyl acetate	Novozym^®^ 435 immobilized *Candida antarctica* lipase B	Packed-bed reactor (continuous) 8 mm i.d. × 200 mm Residence time: 36.5 min Solvent: supercritical CO_2_ Mathematical model Conversion: 95.5%	[42]
Structured lipids from olive oil	Lipozyme^®^ TL-IM immobilized *Thermomyces* *lanuginosus* lipase Lipozyme^®^ RM IM immobilized *Rhizomucor miehei* lipase	Packed-bed reactor (continuous) 2 cm i.d. × 20 cm Residence time: 10.9 and 20 min Solvent: solvent-free Conversion: 70% for 70 h	[43]
Structured lipids from palm-olein	Lipozyme^®^ TL-IM immobilized *Thermomyces* *lanuginosus* lipase	Packed-bed reactor (continuous) 30 mm i.d. × 48 cm Solvent: solvent-free Lipid composition	[44]

**Table 3:** Biocatalytic synthesis of esters with applications in the food industry. Comparison of different reactors.

Ester	Biocatalyst	Characteristics	Reference
Butyl butyrate	Novozym^®^ 435 immobilized *Candida antarctica* lipase B	Batch reactor and membrane reactor (recirculating) Volume BR: 400 mL Volume MR: 175 mL Solvent: supercritical CO_2_ Selectivity: ≥99% after 3 h BR ≥99% after 7 cycles of 6 h MR Better results with MR	[45]
Amyl caprylate	*Candida rugosa* lipase in-lab immobilized on Sepabeads EC-EP	Batch reactor and fluidized bed reactor (recirculating) Volume BR: 10 mL Volume FBR: 80 mL, 10 mm i.d. × 136 mm, residence time: 3.53–0.75 min Solvent: isooctane Water removal: molecular sieves Conversion: ≥99% after 24 h BR 90.2% for 70 h FBR Better results with FBR	[46]
Butyl butyrate	*Thermomyces lanuginosus* lipase (TLL) in-lab immobilized on Immobead 150	Batch reactor, packed-bed reactor, packed-bed reactor with glass beads and fluidized bed reactor (continuous) Volume BR: 10 mL PBR: 10 mm i.d. × 65 mm Solvent: n-hexane Conversion: 21%, 85% and 60% Better results with PBR with glass beads	[47]
Isoamyl acetate	Novozym^®^ 435 immobilized *Candida antarctica* lipase B	Batch reactor and packed-bed reactor (continuous) Volume BR: 100 mL PBR: 3.37 mm i.d. × 0.33 m, residence time: 5.6–11 s Solvent: supercritical CO_2_ Mathematical model (mass transfer, kinetic and reactor design) Better results with PBR	[48]
Sorbitol laurate	10 immobilized lipases 6 lipases in solution	Batch reactor and “tube-reactor” (discontinuous in orbital shaker) Volume BR: 500 mL Conversion: 28% after 48 h BR 50% after 48 h TR	[49]

**Table 4:** Biocatalytic synthesis of esters with applications in the food industry using other reactor configurations.

Ester	Biocatalyst	Characteristics	References
Sugar fatty acid esters	Chirazyme^®^ L-2 immobilized *Candida antarctica* lipase B	Continuous stirred membrane tank reactor Membrane area: 23 cm^2^. Bottom part for the reaction 58 mm i.d. × 2 mm Solvent: ethyl methyl ketone, n-hexane Water removal: azeotrope and membrane evaporation Conversion: 93% after 48 h	[50,51]
Sugar esters	Mycelium-bound *Mucor circinelloides* lipase	Batch microreactor with water activity sensor Volume: 37 mL Solvent: di-n-pentyl and petroleum ethers Water activity influence Conversion: 72% after 20 min	[52]
Alkyl esters	Novozym^®^ 435 Immobilized *Candida antarctica* lipase B	Packed-bed miniaturized reactor (continuous) 1.65 mm i.d. × 30 mm 100 mg lipase, flow rate of 1 µL/min Solvent: n-hexane Conversion: 92% for 2 h	[53]
n-Butyl levulinate	Lipozyme^®^ RM IM immobilized *Rhizomucor miehei* lipase Lipozyme^®^ TL-IM immobilized *Thermomyces* *lanuginosus* lipase Novozym^®^ 435 immobilized *Candida antarctica* lipase B	Packed-bed microreactor (continuous) 3 mm i.d. × 100 mm Residence time: 1–5 min Solvent: tert-butyl methyl ether, 1,4 dioxane, acetonitrile, toluene Conversion: 85% for 25 h (6 runs)	[54]
n-Amyl acetate	*Burkholderia cepacia* lipase in-lab immobilized on a biodegradable polymer	Coated film microreactor (batch and continuous) Volume: 18 mL Solvent: n-hexane Mathematical model (dispersion model) Productivities: 8.16 and 6.54 mmol/g h	[55]
Ascorbyl palmitate	Lipozyme^®^ 435 immobilized *Candida antarctica* lipase B Lipozyme^®^ TL-IM immobilized *Thermomyces* *lanuginosus* lipase Lipozyme^®^ RM IM immobilized *Rhizomucor miehei* lipase Lipozyme^®^ Novo 40086 immobilized *Rhizomucor miehei* lipase Amano lipase PS immobilized *Burkholderia* *cepacia* lipase	Batch reactor: rotating basket, sequential batches Volume: 500 mL Solvent: 2-methyl-2-butanol Water removal: molecular sieves Conversion: 80% each batch (4 batches)	[56]

**Table 5:** Biocatalytic synthesis of esters with applications in the cosmetic industry using tank reactors.

Ester	Biocatalyst	Characteristics	Reference
n-Octyl oleate	Lipozyme^®^ RM IM immobilized *Rhizomucor miehei* lipase	Batch reactor Volume: 102 mL Solvent: supercritical CO_2_ Conversion: 88% after 5 h	[60]
Cetyl palmitate	Novozym^®^ 435 immobilized *Candida antarctica* lipase B *Candida rugosa* lipase in-lab immobilized in MP 1000	Batch reactor Volume: 0.6 L Solvent: solvent-free Water activity measurement and control Conversion: 73% after 192 h	[61]
Ethyl oleate	Novozym^®^ 435 immobilized *Candida antarctica* lipase B	Batch reactor Volume: 250 mL Solvent: solvent-free Kinetic model Conversion: 90% after 5.5 h	[62]
Citronellol laurate	Novozym^®^ 435 immobilized *Candida antarctica* lipase B SP 382 immobilized *Candida antarctica* lipase B Lipozyme^®^ RM IM immobilized *Rhizomucor miehei* lipase	Batch reactor Volume: 100 mL Solvent: n-heptane, supercritical CO_2_ Conversion: 74% after 5 h	[63]
Monolauryl maltose	Novozym^®^ 435 immobilized *Candida antarctica* lipase B	Continuous stirred tank reactor Lipase in a stainless-steel basket Volume: 250 mL Solvent: acetone Water removal: molecular sieves (addition) Successive maltose addition due to insolubility Recycling lauric acid and solvent Productivity: 9.2 g/d L reactor for 10 days	[64]
Oleyl oleate	Novozym^®^ 435 immobilized *Candida antarctica* lipase B	Batch reactor (different agitators) Volume: 2 L Solvent: hexane Kinetic model Conversion: >90% after 1 h (Rushton turbine)	[65]
Kojic acid ricinoleate	Novozym^®^ 435 immobilized *Candida antarctica* lipase B Lipozyme^®^ RM IM immobilized *Rhizomucor miehei* lipase Lipozyme^®^ TL-IM immobilized *Thermomyces* *lanuginosus* lipase	Batch reactor Volume: 500 mL Solvent: solvent-free Water removal: vacuum Conversion: 87.4% after 6 h	[66]
Fatty acid glucose ester	*Candida antarctica* lipase B displaying-*Pichia pastoris* strain GS115/CALB-GCW21-42	Batch reactor Volume: 5 mL, 2 L and 5 L Solvent: different organic solvents Water removal: molecular sieves Conversion: 90% after 96 h	[67]
Kojic acid monooleate	Novozym^®^ 435 immobilized *Candida antarctica* lipase B	Batch reactor Volume: 125 mL Solvent: solvent-free Water removal: atmospheric evaporation Conversion: 44.46% after 5 h	[68]
Cetyl ricinoleate	*Candida antarctica* lipase B in-lab immobilized in Lewatit MonoPlusMP64	Batch reactor Volume: 50 mL and 100 mL Solvent: solvent-free Water removal: atmospheric evaporation and vacuum with dry N_2_ bubbling Conversion: 98% after 3 h	[69]
Myristyl myristate	Novozym^®^ 435 immobilized *Candida antarctica* lipase B	Batch reactor Volume: 50 mL and 100 mL Solvent: solvent-free Water removal: atmospheric evaporation and vacuum with dry N_2_ bubbling Conversion: 99% after 2 h	[70]
n-Butyl palmitate	Fermase CALB 10000 immobilized *Candida antarctica* lipase B	Batch reactor Volume: 250 mL Solvent: solvent-free Water removal: molecular sieves Kinetic model Conversion: 91.25% after 4 h	[71]
Amphiphilic amides	Novozym^®^ 435 immobilized *Candida antarctica* lipase B	Batch reactor Volume: 50 mL Solvent: solvent-free Ethanol removal: vacuum Conversion: 99% after 20 h	[72]
Cetyl fatty acid esters	Novozym^®^ 435 immobilized *Candida antarctica* lipase B	Batch reactor Volume: 100 mL Solvent: solvent-free Water removal: vacuum with dry N_2_ bubbling Conversion: 98.5% after 1.5 h	[73]
Octyl ethanoate	Novozym^®^ 435 immobilized *Candida antarctica* lipase B	Batch reactor with ultrasound Volume: 50 mL Solvent: solvent-free Kinetic model Conversion: 97.31% after 20 min	[74]
Oleic acid sugar esters	Novozym^®^ 435 immobilized *Candida antarctica* lipase B	Batch reactor Volume: 100 mL Solvent: solvent-free Water removal: molecular sieves Conversion: 96.6% after 6 days	[75]
Hexyl acetate	Lipozyme^®^ RM IM immobilized *Rhizomucor miehei* lipase	Batch reactor with ultrasound Volume: 50 mL Solvent: hexane Conversion: 85% after 4 h	[76]
2-Phenylethyl acetate	Novozym^®^ 435 immobilized *Candida antarctica* lipase B	Batch reactor Volume: 50 mL Solvent: hexane Kinetic model Conversion: 95.42% after 2 h	[77]
Geranyl acetate	Novozym^®^ 435 immobilized *Candida antarctica* lipase B	Batch reactor Volume: 50 mL Solvent: hexane Kinetic model Conversion: 98.4% after 160 min	[78]
Butyl stearate ethyl stearate	Novozym^®^ 435 immobilized *Candida antarctica* lipase B	Batch and fedbatch reactors Volume: 250 mL Solvent: solvent-free Conversion: 92% after 24 h	[79]
Spermaceti analogue	Lipozyme^®^ RM IM immobilized *Rhizomucor miehei* lipase CalB immo Plus immobilized *Candida antarctica* lipase B	Batch reactor Volume: 100 mL Solvent: solvent-free Water removal: vacuum with dry N_2_ bubbling Conversion: 98% after 2 h	[80]
n-Butyl palmitate	Fermase CALB 10000 immobilized *Candida antarctica* lipase B	Batch reactor with ultrasound Volume: 100 mL Solvent: solvent-free Water removal: molecular sieves Kinetic model Conversion: 96.6% after 50 min	[81]
Palm oil esters	Lipozyme^®^ RM IM immobilized *Rhizomucor miehei* lipase Lipozyme^®^ TL-IM immobilized *Thermomyces* *lanuginosus* lipase	Batch reactor (scale-up) Volume: 2 L, 15 L, and 300 L Solvent: n-hexane and solvent-free Conversion: >90% after 3 h	[82]
2-Ethylhexyl palmitate	Fermase CALB 10000 immobilized *Candida antarctica* lipase B	Batch reactor with ultrasound Volume: 50 mL Solvent: solvent-free Conversion: 96.56% after 2 h	[83]
Cetyl caprate	Fermase CALB 10000 immobilized *Candida antarctica* lipase B	Batch reactor Volume: 50 mL Solvent: solvent-free Kinetic model Conversion: 95% after 80 min	[84]
Spermaceti analogue	*Candida antarctica* lipase B in-lab immobilized in different supports	Batch reactor Volume: 100 mL Solvent: solvent-free Economic study Water removal: vacuum with dry N_2_ bubbling Conversion: >90% after 1 h	[85]
Cetyl oleate	Fermase CALB 10000 immobilized *Candida antarctica* lipase B	Batch reactor with ultrasound Volume: 50 mL Solvent: solvent-free Conversion: 97.5% after 20 min	[86]
Isoamyl and cinnamyl acetate	Lyophilized mycelium of *Aspergillus oryzae*	Continuous stirred tank membrane reactor Volume: 200 mL Residence time: 500 min Solvent: n-heptane Conversion: 98% for 10 days	[87]
Fatty acid ascorbyl esters	Novozym^®^ 435 immobilized *Candida antarctica* lipase B	Batch reactor Volume: 100 mL Solvent: organic solvent Kinetic model Conversion: high conversion depending on the fatty acid	[88]
2-Ethylhexyl stearate	Fermase CALB 10000 immobilized *Candida antarctica* lipase B	Batch reactor with ultrasound Volume: 50 mL Solvent: solvent-free Conversion: 95.87% after 3 h	[89]
Benzyl acetate	Novozym^®^ 435 immobilized *Candida antarctica* lipase B Novozym^®^ 40086 immobilized *Rhizomucor miehei* lipase Lipozyme^®^ TL-IM immobilized *Thermomyces* *lanuginosus* lipase	Batch reactor Volume: 50 mL Solvent: n-hexane and n-heptane Kinetic model Conversion: >90% after 2.5 h	[90]
2-Ethylhexyl palmitate and stearate	Novozym^®^ 435 immobilized *Candida antarctica* lipase B Novozym^®^ 40086 immobilized *Rhizomucor miehei* lipase	Batch reactor Volume: 100 mL Solvent: solvent-free Water removal: vacuum with dry N_2_ bubbling Conversion: 98% after 45 min	[91]
Neopentyl glycol diheptanoate	Novozym^®^ 435 immobilized *Candida antarctica* lipase B	Batch and fed-batch reactors Volume: 50 mL Solvent: solvent-free Water removal: atmospheric evaporation Conversion: 95% after 6 h	[92]
Spermaceti analogue	Novozym^®^ 435 immobilized *Candida antarctica* lipase B Lipozyme^®^ TL-IM immobilized *Thermomyces* *lanuginosus* lipase	Batch reactor Volume: 100 mL Solvent: solvent-free Economic study Water removal: vacuum with dry N_2_ bubbling Conversion: >97.5% after 2 h	[93]
Isopropyl palmitate	*Penicillium camemberti* lipase in-lab immobilized on magnetized poly(styrene- codivinylbenzene)	Batch reactor Volume: 280 mL Solvent: heptane Kinetic model Conversion: 85.65% after 12 h	[94]
Decyl oleate	Fermase CALB 10000 immobilized *Candida antarctica* lipase B	Batch reactor with ultrasounds Volume: 50 mL Solvent: solvent-free Kinetic model Conversion: 97.14% after 25 min	[95]
2-Ethylhexyl 2-methylhexanoate	Novozym^®^ 435 immobilized *Candida antarctica* lipase B	Batch reactor Volume: 50 mL Solvent: solvent-free Economic study and green metrics Water removal: atmospheric evaporation Conversion: 99.74% after 5 h	[96]
Spermaceti analogue	Novozym^®^ 435 immobilized *Candida antarctica* lipase B Lipozyme^®^ RM IM immobilized *Rhizomucor miehei* lipase Lipozyme^®^ TL-IM immobilized *Thermomyces* *lanuginosus* lipase CalB immo Plus immobilized *Candida antarctica* lipase B	Batch reactor Volume: 100 mL Solvent: solvent-free Kinetic model Water removal: vacuum with dry N_2_ bubbling Conversion: >90% after 1 h	[97]
Neopentyl glycol dicaprylate/ dicaprate	Lipozyme^®^ 435 immobilized *Candida antarctica* lipase B	Batch and fed-batch reactors Volume: 50 mL Solvent: solvent-free Economic study and green metrics Water removal: atmospheric evaporation Conversion: 92.5% after 6 h	[98]
Neopentyl glycol dilaurate	Novozym^®^ 435 immobilized *Candida antarctica* lipase B Novozym^®^ 40086 immobilized *Rhizomucor miehei* lipase	Batch reactor Volume: 50 mL Solvent: solvent-free Economic study and green metrics Water removal: atmospheric evaporation Conversion: >90% after 6 h	[99]
Spermaceti analogue	*Candida antarctica* lipase B in-lab immobilized in Purolite^®^ Lifetech™ ECR8285	Batch reactor Volume: 100 mL Solvent: solvent-free Water removal: vacuum with dry N_2_ bubbling Conversion: 97% after 1 h Production plant simulation using aspenONE suite v10	[100]
Panthenyl monoacyl ester	Novozym^®^ 435 immobilized *Candida antarctica* lipase B	Batch reactor Volume: 500 mL Solvent: solvent-free (eutectic mixture) Green metrics Conversion: 87−95% after 6 h	[10]
Octyl oleate	*Candida antarctica* lipase B in-lab immobilized on magnetic poly(STY-EGDMA) particles	Batch reactor Volume: 100 mL Solvent: solvent-free Kinetic model Conversion: 57% after 24 h	[102]

**Table 6:** Biocatalytic synthesis of esters with applications in the cosmetic industry using tubular reactors.

Ester	Biocatalyst	Characteristics	Reference
Mono-, di-, and triacyglycerols from (poly)unsaturated fatty acids	Chirazyme^®^ L-9 immobilized *Mucor miehei* lipase	Packed-bed reactor (continuous) 0.32 cm i.d. × 20 cm 0.47 cm i.d. × 8.9 cm 0.63 cm i.d. × 5 cm 0.79 cm i.d. × 3.2 Residence time: 15 min Solvent: hexane, 2-propanol, ethyl acetate, formic acid Conversion: 80–90% for 12 days	[105]
Cetyl palmitate	SP 435 immobilized *Candida antarctica* lipase B	Packed-bed reactor (continuous) Silicone and PVC tube: 3 mm i.d. Flow rate: 0.005 g/min Productivity: 7.2 g/day Conversion: 99.1% for 7 days	[106]
Feruloylated monoacyl- and diacyl glycerols	Novozym^®^ 435 immobilized *Candida antarctica* lipase B	Packed-bed reactor (recirculating) 2.5 cm i.d. × 30 cm Flow rate: 2 mL/min Solvent: solvent-free Water removal: molecular sieves Conversion: 60% after 140 h	[107]
Hexyl laurate	Lipozyme^®^ IM-77 immobilized *Rhizomucor miehei* lipase	Packed-bed reactor (continuous) 0.25 cm i.d. × 25 cm Residence time: 0.43 min Solvent: n-hexane Conversion: 97%	[108]
Hexyl laurate	Lipozyme^®^ IM-77 immobilized *Rhizomucor miehei* lipase	Packed-bed reactor (continuous) 0.25 cm i.d. × 25 cm Flow rate: 0.55 mL/min Solvent: solvent-free Production rate: 87.44 μmol/min	[109]
Citronellyl malonate	*Candida rugosa* lipase in-lab immobilized on Amberlite MB-1	Packed-bed reactor (continuous) 1.2 cm i.d. × 24 cm Flow rate: 1 mL/min Solvent: *iso*-octane Kinetic model Water removal: molecular sieves Conversion: 90% (steady state after 180 min)	[110]
Lard-based ascorbyl esters	Novozym^®^ 435 immobilized *Candida antarctica* lipase B	Packed-bed reactor (continuous) 2 cm i.d. × 10 cm or 25 cm Flow rate: 0.07 mL/min Solvent: *tert*-amyl alcohol Water removal: molecular sieves Conversion: 50.50%	[111]
Feruloyl soy glycerides	Novozym^®^ 435 immobilized *Candida antarctica* lipase B	Packed-bed reactor (continuous, pilot scale) Four 304-stainless steel columns 9.8 cm i.d. × 132 cm Flow rate: 2.5 mL/min Solvent: solvent-free Conversion: 65% for 4.5 months	[112]
Dibehenyl adipate Dibehenyl sebacate	Lipozyme^®^ RM IM immobilized *Rhizomucor miehei* lipase Lipozyme^®^ TL-IM immobilized *Thermomyces* *lanuginosus* lipase SP 435 immobilized *Candida antarctica* lipase B NS40013 immobilized *Candida antarctica* lipase B	Packed-bed reactor (continuous) 1 in i.d. × 12 in Flow rate: 3 mL/min Solvent: isooctane Water removal: vacuum Conversion: 89% and 91% for 5 h; 20 reuses	[113]
2-Ethylhexyl palmitate	*Candida* sp. 99–125 lipase in-lab immobilized on a fabric membrane	Packed-bed reactor (recirculating) 40, 60, 90 mm i.d. × 630, 280, 124 mm Residence time: 160 s Solvent: solvent-free Study of H/D influence on conversion Water removal: molecular sieves Conversion: 95% for 300 h (30 batches)	[114]
Polyglycerol fatty acid esters	Lipozyme^®^ 435 immobilized *Candida antarctica* lipase B	Bubble column reactor (batches) Volume: 2 L Solvent: solvent-free Water removal: vacuum and N_2_ bubbling Conversion: 95.82% for 4.25 h (10 batches)	[115]
Eugenyl acetate	Lipozyme^®^ TL-IM immobilized *Thermomyces* *lanuginosus* lipase	Packed-bed reactor (continuous) 15 mm i.d. × 55 mm Residence time: 55, 7, and 4 min Solvent: solvent-free Conversion: 93.1%	[116]
Soybean-free fatty acids ethyl esters	Novozym^®^ 435 immobilized *Candida antarctica* lipase B Lipozyme^®^ TL-IM immobilized *Thermomyces* *lanuginosus* lipase	Packed-bed reactor with ultrasound (continuous) 14.5 mm i.d. × 171 mm Flow rate: 2.5 mL/min Conversion: 95% at 6 min residence time	[117]
Fructose stearate	*Rhizomucor miehei* lipase in-lab immobilized into chicken eggshells	Packed-bed reactor (continuous) 10 mm i.d.× 90 mm Flow rate: 0.074 mL/min Solvent: ethanol Product concentration: 7.252 × 10^−1^ mol/L	[118]
Geranyl butyrate	*Candida rugosa* lipase in-lab immobilized on different resins	Fluidized bed reactor (recirculating) 1.4 cm i.d. × 17 cm Flow rate: 0.07 mL/min Residence time: 4.7 h Solvent: n-heptane Water removal: molecular sieves Conversion: 77% for 12 h	[119]
2-Ethylhexyl oleate	*Candida antarctica* lipase in-lab immobilized on STY-DVB-M particles	Fluidized bed reactor (continuous with recirculation) 15 mm i.d. × 202 mm Residence time: 6, 12, and 18 h Solvent: solvent-free Mathematical model (kinetic and mass transfer) Conversion: 48.24% for 8 days	[120]
2-Ethylhexyl oleate	*Candida antarctica* lipase in-lab immobilized on STY-DVB-M particles	Packed-bed reactor (continuous) 11 mm i.d. × 166 mm Residence time: 3, 6, and 12 h Solvent: solvent-free Kinetic model Conversion: 60% for 16 days	[121]
2-Ethylhexyl oleate	Novozym^®^ 435 immobilized *Candida antarctica* lipase B	Packed-bed reactor (semicontinuous) 12 mm i.d. × 300 mm Flow rate: 1.5 mL/min Solvent: solvent-free Water removal: molecular sieves Economic study and process plant simulation Conversion: >95% for 12 cycles × 720 h each	[122]
2-Phenylethyl acetate	Novozym^®^ 435 immobilized *Candida antarctica* lipase B	Packed-bed reactor (continuous) 0.46 cm i.d. × 25 cm Flow rate: 1, 3, and 5 mL/min Solvent: solvent-free Conversion: 100% for 10 min (lower flow rate)	[123]
Glucose mono decanoate	Novozym^®^ 435 immobilized *Candida antarctica* lipase B	Packed-bed reactor (continuous recycling glucose) XK16 column from Cytiva Flow rate: 0.5 mL/min Residence time: 13 min Productivity: 1228 µmol/L h	[124]
Monoacyl glycerols of Babassu oil	*Burkholderia cepacia* lipase in-lab immobilized on SiO_2_–PVA particles	Packed-bed reactor (continuous) 15 mm i.d. × 55 mm Residence time: 9.8 h Mathematical model (mass transfer) Productivity: 52.3 mg/g h	[125]
2-Ethylhexyl oleate	*Candida antarctica* lipase in-lab immobilized on STY-DVB-M particles	Fluidized bed reactor magnetically stabilized (continuous recycling substrate) 15 mm i.d. × 202 mm Flow rate: 0.044 mL/min Solvent: solvent-free Residence time: 12 h Kinetic model Conversion: 55.63% for 16 days	[126]

**Table 7:** Biocatalytic synthesis of esters with applications in the cosmetic industry. Comparison of different reactors.

Ester	Biocatalyst	Characteristics	Reference
α-Butylglucoside linoleate	Chirazyme^®^ L-2 C2 immobilized *Candida antarctica* lipase B Chirazyme^®^ L-9 immobilized *Mucor miehei* lipase	Batch reactor and packed-bed reactor (recirculating with mixing tank) BR: rotary evaporator (Büchi, R-114) PBR: 20 cm i.d. × 150 cm, flow rate: 4.5 mL/min Solvent: decane Water removal: vacuum Conversion: >90% for >5 cycles × 70 h each Better results with PBR and Chirazyme L-9	[127]
Myristyl myristate	Novozym^®^ 435 immobilized *Candida antarctica* lipase B	Batch reactor, packed-bed reactor, and bubble column reactor Solvent: solvent-free Water removal: vacuum Mathematical model (kinetic and mass transfer) Conversion: 99.6% after 5.5 h bubble column reactor after 17 h packed-bed reactor after 24 h batch reactor Better results with bubble column reactor	[128]
Geranyl acetate	Novozym^®^ 435 immobilized *Candida antarctica* lipase B	Batch reactor, packed-bed reactor, and PBR series PBR: 3.2 mm i.d. × different lengths Solvent: supercritical CO_2_ and supercritical ethane Mathematical model (kinetic and reactor design) Batter results with two reactors in series	[129]
Geranyl butyrate	*Candida rugosa* lipase in-lab immobilized on Sepabeads^®^ EC-EP, Sepabeads^®^ EC-HA and Purolite^®^ A-109	Batch reactor and fluidized bed reactor (recirculating) Volume BR: 100 mL PBR: 10 mm i.d. × 136 mm Solvent: isooctane Mathematical model (hydrodynamic) Conversion: >99.9% after 48 h BR 78.9% after 10 h FBR Better results with FBR	[130]
Cetyl oleate	Fermase CALB 10000 immobilized *Candida antarctica* lipase B	Batch reactor and batch reactor with ultrasound Volume BR: 250 mL Volume BR ultrasound: 50 mL Solvent: solvent-free Water removal: molecular sieves Kinetic model Conversion: 95.96% after 2 h BR 95.96% after 30 min BR with ultrasound Better results with BR (stirred) with ultrasound	[131]
Ascorbyl oleate	*Candida antarctica* lipase in-lab immobilized on Purolite^®^ MN102	Batch reactor and fluidized bed reactor (recirculating) Volume BR: 5 mL FBR: 9 mm i.d. × 136 mm Solvent: tert-butanol Water removal: molecular sieves Mathematical model (kinetic and hydrodynamic) Better results with FBR (no damage of particles)	[132]
Kojic acid derivatives with fatty acids	Novozym^®^ 435 immobilized *Candida antarctica* lipase B Lipozyme^®^ RM IM immobilized *Rhizomucor miehei* lipase Lipozyme^®^ TL-IM immobilized *Thermomyces* *lanuginosus* lipase	Batch reactor and fluidized tank reactor Volume BR: 100 mL FBR: The same BR sparged with air Solvent: solvent-free Better results with BR	[133]
Isoamyl laurate	Five microbial lipases In-lab immobilized on epoxy-polysiloxane-hydroxyethylcellulose and styrene-divinylbenzene	Batch reactor and packed-bed reactor (continuous) Volume BR: 20 mL PBR: 15 mm i.d. × 55 mm; flow rate: 1.8 mL/h; residence time: 3.12 h Solvent: solvent-free Conversion: 81.26% after 24 h BR 0.8 mol/L h PBR for 168 h Better results with PBR	[134]
Polyglycerol-10 laurate Polyglycerol-10 caprylate	Novozym^®^ 435 immobilized *Candida antarctica* lipase B	Batch reactor (with mechanical agitation and bubbling) and fluidized bed reactor (batch operation) Volume BR: Duran bottle (unknown volume) FBR: 20 mm i.d. × 35 cm Solvent: solvent-free Water removal: dry N_2_ bubbling Conversion: ≈100% after 20 h and 22 h Better results with N_2_ bubbling	[135]

**Table 8:** Biocatalytic synthesis of esters with applications in the cosmetic industry using other reactor configurations.

Ester	Biocatalyst	Characteristics	Reference
Hexyl acetate	*Fusarium solani pisi* cutinase cloned and expressed in *Escherichia coli*	Membrane reactor stainless-steel monochannel ultrafiltration module (continuous) Volume: 100 mL Membrane area: 38 cm^2^ Homogenization achieved through partial recirculation Flow rate: 0.1 mL/min Solvent: iso-octane (reversed micelles) Mathematical model (reactor design) Good performance of the MR	[136]
Isoamyl acetate	Novozym^®^ 435 immobilized *Candida antarctica* lipase B	Packed-bed microreactor (microchannel, continuous) 1 cm width × 450 µm height × 75 mm length Solvent: ionic liquid Conversion: 92% in 15 min (multiple runs for 2 weeks)	[137]
Eugenyl esters	Novozym^®^ 435 immobilized *Candida antarctica* lipase B Lipozyme^®^ RM IM immobilized *Rhizomucor miehei* lipase	Packed-bed microreactor (continuous) 0.5 cm i.d. × 5 cm Solvent: solvent-free Conversion: 82% N435 and 90% RM IM for 26 h (acetate)	[138]

## Data Availability

Not applicable.

## Abbreviations

BR	batch reactor
CAGR	compound annual growth rate
CALB	*Candida antarctica* lipase B
CRL	*Candida rugosa* lipase
DES	deep eutectic solvent
FBR	fluidized bed reactor
GRAS	generally recognized as safe
PBR	packed-bed reactor
WOS	Web of Science
